# Vascular dysfunction in aged mice contributes to persistent lung fibrosis

**DOI:** 10.1111/acel.13196

**Published:** 2020-07-21

**Authors:** Nunzia Caporarello, Jeffrey A. Meridew, Aja Aravamudhan, Dakota L. Jones, Susan A. Austin, Tho X. Pham, Andrew J. Haak, Kyoung Moo Choi, Qi Tan, Adil Haresi, Steven K. Huang, Zvonimir S. Katusic, Daniel J. Tschumperlin, Giovanni Ligresti

**Affiliations:** ^1^ Department of Medicine Boston University School of Medicine Boston MA USA; ^2^ Department of Physiology & Biomedical Engineering Mayo Clinic Rochester MN USA; ^3^ Department of Anesthesiology and Molecular Pharmacology and Experimental Therapeutics Mayo Clinic Rochester MN USA; ^4^ Department of Internal Medicine University of Michigan Medical School Ann Arbor MI USA

**Keywords:** aging, eNOS, fibroblast activation, lung fibrosis, vascular dysfunction

## Abstract

Idiopathic pulmonary fibrosis (IPF) is a progressive disease thought to result from impaired lung repair following injury and is strongly associated with aging. While vascular alterations have been associated with IPF previously, the contribution of lung vasculature during injury resolution and fibrosis is not well understood. To compare the role of endothelial cells (ECs) in resolving and non‐resolving models of lung fibrosis, we applied bleomycin intratracheally to young and aged mice. We found that injury in aged mice elicited capillary rarefaction, while injury in young mice resulted in increased capillary density. ECs from the lungs of injured aged mice relative to young mice demonstrated elevated pro‐fibrotic and reduced vascular homeostasis gene expression. Among the latter, *Nos3* (encoding the enzyme endothelial nitric oxide synthase, eNOS) was transiently upregulated in lung ECs from young but not aged mice following injury. Young mice deficient in eNOS recapitulated the non‐resolving lung fibrosis observed in aged animals following injury, suggesting that eNOS directly participates in lung fibrosis resolution. Activation of the NO receptor soluble guanylate cyclase in human lung fibroblasts reduced TGFβ‐induced pro‐fibrotic gene and protein expression. Additionally, loss of eNOS in human lung ECs reduced the suppression of TGFβ‐induced lung fibroblast activation in 2D and 3D co‐cultures. Altogether, our results demonstrate that persistent lung fibrosis in aged mice is accompanied by capillary rarefaction, loss of EC identity, and impaired eNOS expression. Targeting vascular function may thus be critical to promote lung repair and fibrosis resolution in aging and IPF.

## Graphical abstract

Bleomycin‐induced lung injury promotes transient fibrosis accompanied by increased capillary density in young mice. In contrast, persistent fibrosis, capillary rarefaction, loss of endothelial cell identity, and reduction of Nos3 are observed in aged mice. eNOS/NO signal is an important driver of fibroblast quiescence and fibrosis resolution, that is lost with aging. Lung vascular bed plays a critical role during lung repair and fibrosis resolution.

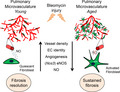

## 1 INTRODUCTION

Idiopathic pulmonary fibrosis (IPF) is the most common idiopathic interstitial pneumonia, and its incidence and prevalence greatly increase with age (Lederer & Martinez, 2018). IPF prognosis is poor with a median survival of 2‐3 years after diagnosis and a mortality rate higher than most common cancers (Mora, Rojas, Pardo, & Selman, 2017). Abnormal wound healing, excessive scarring, and loss of gas exchange function are cardinal features of IPF, and current therapeutic options are limited and not able to fully reverse established disease (Somogyi et al., 2019). It has been hypothesized that the limited regenerative capacity of the aged lung may greatly influence lung repair and ultimately fibrosis resolution (Meiners, Eickelberg, & Konigshoff, 2015), but the role of the lung vasculature in fibrosis and repair of the aging lung is not well studied.

Pulmonary microvasculature is abundant in mature lung and plays a critical role in mediating gas exchange (Aird, 2007). In addition to carrying blood, endothelial cells actively release angiocrine factors that have been shown to regulate both alveogenesis during mouse lung development as well as lung regeneration in adult animals (Ding et al., 2011), making these cells important regulators of lung homeostasis. Intriguingly, endothelial dysfunction increases with advancing aging and multiple lines of research have established that vascular aging is a critical step in the development of numerous chronic disorders including chronic lung diseases (Polverino, Celli, & Owen, 2018; Seals, Jablonski, & Donato, 2011). These findings suggest that targeting specific aging‐associated endothelial alterations may represent a therapeutic strategy to promote lung repair and halt disease progression.

Aberrant vascular remodeling is a previously noted feature in the pathogenesis of IPF, and increased capillary irregularities including vessel dilatation and loss of microvasculature have been observed in IPF lungs (Barratt & Millar, 2014; Mlika, Bacha, Braham, & El Mezni, 2016; Renzoni, 2004). Although these vascular abnormalities have been well documented, whether they are important drivers of disease progression still remains debated. Thus, understanding the roles lung vascular remodeling plays in lung repair and fibrosis in young and aged animal models may provide important insights in the pathogenesis of IPF.

Prior work with mouse models have showed that lung microvasculature undergoes extensive remodeling following bleomycin‐induced lung injury (Kato et al., 2018). Inhibiting key angiogenic pathways in endothelial cells (ECs) during the early injury and inflammatory phase post‐bleomycin delivery limits fibroblast activation and reduces collagen deposition (Dang et al., 2017; Wan et al., 2013). While these studies suggest that ECs positively support fibrogenic responses during the early phase post‐injury, they do not provide specific insights on the role of the vasculature during later phases of lung repair and fibrosis resolution. Given the important role of vascular ECs in homeostasis and regeneration of multiple organs, including lungs (DeLisser et al., 2006; Ding et al., 2010; Maeda et al., 2002; Nolan et al., 2013), we reasoned that ECs from the lung vascular bed may play an important role during fibrosis resolution by limiting fibrogenic milieus and reestablishing a functional alveolar niche.

We have recently demonstrated that lung fibroblast activation following bleomycin challenge is transient in young mice but more persistent in aged ones (Caporarello et al., 2019). Similarly, other groups have shown that fibrosis is persistent in aged mice and resolution is markedly impaired relative to young mice (Hecker et al., 2014; Podolsky et al., 2020; Torres‐Gonzalez et al., 2012; Xu et al., 2009). Thus, in this work, we have taken advantage of the resolving and non‐resolving nature of fibrosis in young and aged mice to compare vascular remodeling and lung endothelial cell behavior associated with these divergent outcomes. We have found that injured lungs from young and aged mice displayed divergent vascular remodeling, with dramatic capillary rarefaction observed in aged mice following bleomycin injury. Gene expression analysis of freshly isolated lung ECs identified profound transcriptional changes in these cells after injury in young and aged mice, including a marked reduction of endothelial cell markers and an increased expression of pro‐fibrotic and inflammatory markers in the ECs from aged mice. In addition, we identified endothelial nitric oxide synthase (eNOS) as an important player during the resolution phase of lung fibrosis that fails to increase in aged mice. Co‐culture experiments demonstrated that lung ECs restrained fibroblast activation and loss of eNOS in vascular cells failed to promote fibroblast deactivation.

Hence, our data shed new light on the pulmonary vasculature as an important regulator of lung fibrosis resolution and identified altered transcriptional responses in lung endothelial cells during aging that may be targeted to promote fibrosis resolution.

## 2 RESULTS

### 2.1 Vascular rarefaction accompanies persistent fibrosis in aged mice

Numerous studies have shown that bleomycin‐induced lung fibrosis in young mice resolves over time (Tashiro et al., 2017). Using Col1α1‐GFP transgenic mice in combination with fluorescence‐activated cell sorting (FACS) analysis, we have previously shown a transient but reversible expansion of high GFP+ lung fibroblasts from young mice following delivery of a single dose of bleomycin to the lung, whereas fibroblasts isolated from the lungs of aged mice exhibited persistent expansion of the high GFP+ population (Caporarello et al., 2019).

**FIGURE 1:**
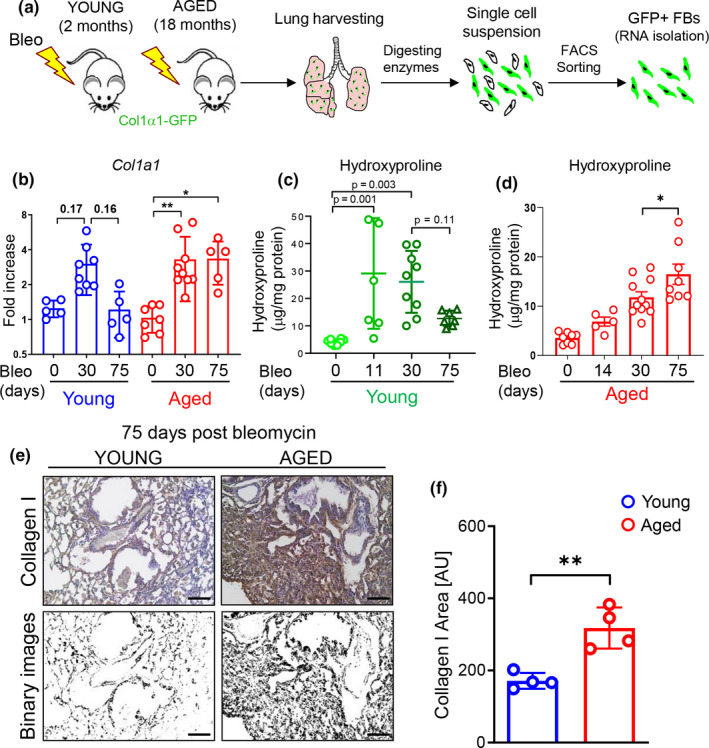
Delayed fibrosis resolution in aged mice following bleomycin challenge. (a) Young and aged mice were exposed to bleomycin and sacrificed after 30 and 75 days. Lungs were harvested and prepared for FACS sorting. (b) *Col1a1* transcriptional analysis of FACS‐sorted GFP+/CD31−/CD45−/EpCAM− lung fibroblasts isolated from young and aged animals after bleomycin‐induced injury (young sham, *N* = 5; young 30 days, *N* = 8; young 75 days, *N* = 5; aged sham, *N* = 7; aged 30 days, *N* = 9; aged 75 days, *N* = 5). (c) Hydroxyproline assay was used to evaluate collagen deposition in the lungs (young sham, *N* = 8; young 14 days, *N* = 7; young 30 days, *N* = 9; young 75 days, *N* = 7). Data passed Kolmogorov–Smirnov normality test, are expressed as mean ± *SD*, and analyzed using one‐way analysis of variance (followed by Tukey's post hoc test). (d) Hydroxyproline assay was used to evaluate collagen deposition in the lungs (aged sham, *N* = 8; aged 14 days, *N* = 5; aged 30 days, *N* = 11; aged 75 days, *N* = 8). Data passed Kolmogorov–Smirnov normality test, are expressed as mean ± *SD*, and analyzed using one‐way analysis of variance (followed by Tukey's post hoc test). (e, f). Representative immunohistochemistry images and quantification of Collagen I by automated image analysis (young 75 days, *N* = 4; aged 75 days, *N* = 4). Data are non‐normally distributed, are expressed as median and IQR, and analyzed using non‐parametric Mann–Whitney test (*p < 0.05; **p < 0.01).

Here, we expanded on this approach to compare lung fibroblast collagen gene expression at timepoints associated with fibrosis resolution in young mice (30 and 75 days post‐bleomycin) and compared responses in young (2 months) and aged mice (18 months). Analysis of body weight and survival curves showed no significant differences for these parameters between the two groups (Figure S1). As shown in Figure 1b, lung fibroblasts isolated from both young and aged mice showed comparable elevation of *Col1a1* transcripts at day 30 following bleomycin treatment. However, while *Col1a1* expression trended downwards in lung fibroblasts from young mice at 75 days post‐bleomycin, its expression remained elevated in lung fibroblasts from aged animals at the same timepoint. These results concur with our prior observation that bleomycin‐induced lung fibrosis in young mice resolves over time with hydroxyproline content peaking at 11 days and returning to baseline at 75 days post‐bleomycin (Caporarello et al., 2019). In contrast, here we show that in aged mice, lung fibrosis continues to increase out to day 75 following a single dose of bleomycin (Figure 1d). Histological analysis confirmed increased collagen deposition in the lungs of aged mice compared to those from young animals at 75 days post‐bleomycin (Figure 1e,f). These data confirm that lungs from young animals spontaneously resolve from bleomycin‐induced fibrosis while lungs from aged animals maintained elevated collagen levels.

**FIGURE 2:**
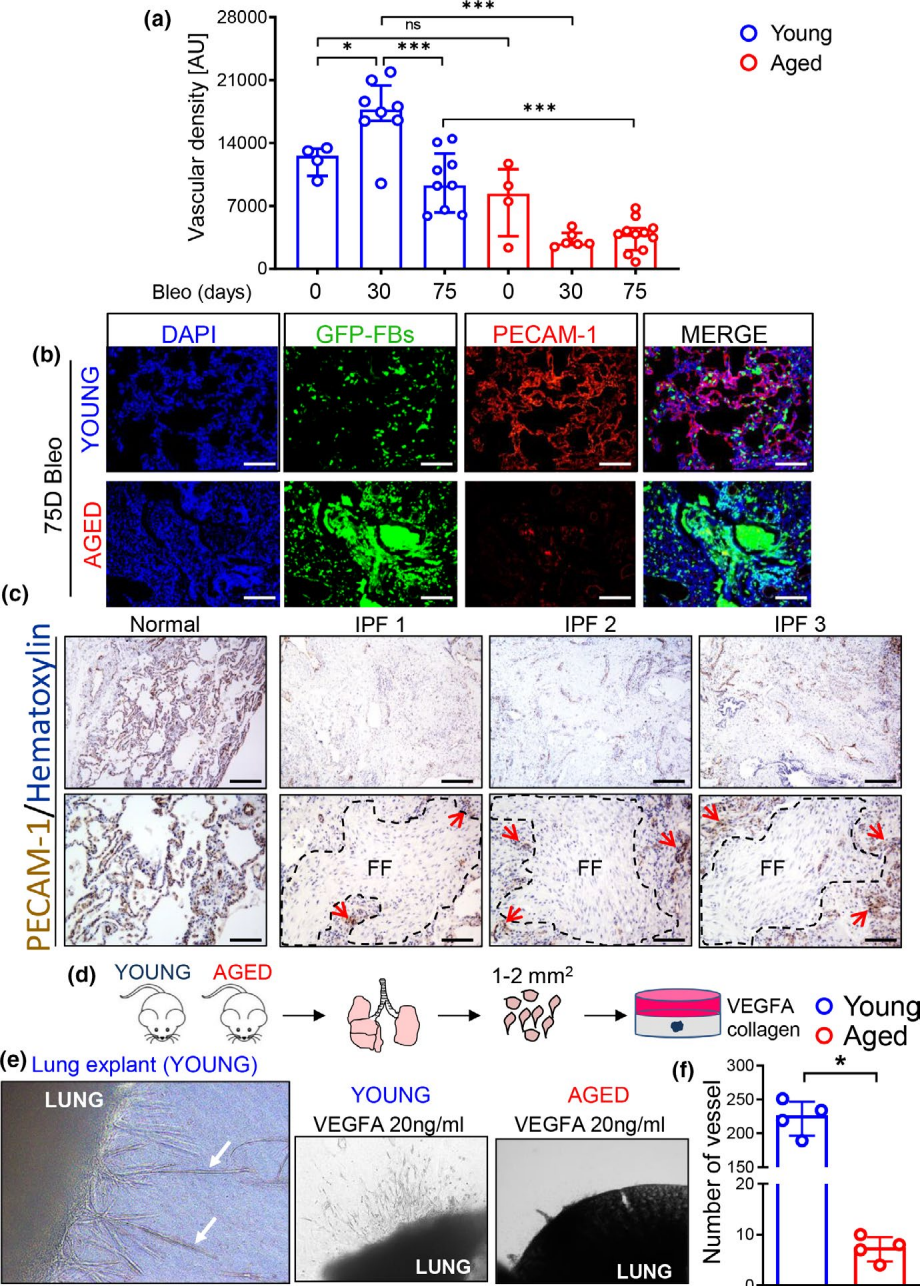
Vascular rarefaction accompanies persistent fibrosis in aged mice challenged with bleomycin. (a) Quantification of vascular density by automated image analysis (young sham, *N* = 4; young 30 days, *N* = 8; young 75 days, *N* = 9; aged sham, *N* = 4; aged 30 days, *N* = 6; aged 75 days, *N* = 11). Data passed Shapiro–Wilk normality test, are expressed as mean ± *SD*, and analyzed using one‐way analysis of variance (followed by Tukey's post hoc test). (b) Representative IF images of mouse lung tissue stained with PECAM‐1 antibody. Scale bars: 100 μm. (c) Immunostaining of human tissue derived from normal or IPF lung for PECAM‐1 counterstained with hematoxylin. Magnifications: upper row, 4X, scale bars: 250 μm, lower row, 10X, scale bars: 100 μm. FF = fibroblastic foci. Arrows show areas occupied by microvessels in regions bordering FF. (d) Schematic for ex vivo lung tissue culture. Pieces of lungs from young and aged mice were embedded in collagen for 7 days in presence of 20 ng/ml VEGFA. (e) Collagen gel culture of lung explants derived from young and aged mice. (f) Vessel counts demonstrate reduction of sprouting outgrowth in aged mice. Data are non‐normally distributed, are expressed as median and IQR, and analyzed using non‐parametric Mann–Whitney test (*p < 0.05; ***p < 0.001).

Because aging‐induced functional and structural alterations of the microcirculation contribute to the pathogenesis of a range of age‐related diseases (Scioli, Bielli, Arcuri, Ferlosio, & Orlandi, 2014), we sought to investigate changes to the pulmonary vasculature in aged mice following lung injury. To evaluate vascular changes that are associated with sustained fibrosis, we immunostained bleomycin‐treated lung tissues from young and aged mice with an antibody against the endothelial cell marker PECAM‐1 followed by an automated vascular density analysis. While we did not observe significant differences in the vascular density between young and aged animals in the absence of injury, lungs from aged mice exhibited significant reduction of vessel density at 30 and 75 days post‐bleomycin relative to injured young mice (Figure 2a,b). These observations resemble the vascular regression we observed within the fibroblastic foci (FF) of human lung tissue from IPF patients (Figure 2c) and are in agreement with previous analyses of IPF fibroblastic foci described in literature (Cosgrove et al., 2004). In contrast, lungs of young mice showed a significant but transient increase in vessel density at the same timepoints (Figure 2a,b). These data demonstrated that persistent lung fibrosis in aged mice is accompanied by a reduced vascular density, suggesting that aging may alter lung endothelial cell responses that contribute to the divergent lung remodeling observed in young and aged mice.

### 2.2 Aged mice lungs showed impaired angiogenic capacity *ex vivo*

Angiogenesis, the growth of new blood vessels from pre‐existing ones, plays a critical role in tissue injury responses, and abnormal/limited angiogenesis has been shown to contribute to the pathogenesis of numerous chronic disorders (Carmeliet, 2003). Thus, we reasoned that the reduced vascular density we observed in aged mice may be the consequence of an altered angiogenic response in lung ECs from these mice. In order to evaluate the angiogenic capacity of lung ECs from young and aged mice, we generated lung explant cultures by embedding freshly cut lung tissues in collagen matrices. In this model, angiogenesis is triggered both as a consequence of the injury associated with the dissection procedure (Ligresti, Aplin, Zorzi, Morishita, & Nicosia, 2011) as well as in response to the presence of exogenous vascular endothelial growth factor A (VEGFA) (Dang et al., 2017). The growth of vessel sprouts from the lung explants was monitored for 7 days and then quantified by counting the number of sprouts in each lung explant under the microscope. As shown in Figure 2e,f, while lung explants from young mice showed numerous vessel sprouts, those harvested from aged animals exhibited a reduced number of vessel sprouts. These results demonstrate that lungs from aged mice have a limited angiogenic capacity compared to young animals, and suggest that aged ECs may differ in their capacity to respond to injury and angiogenic stimuli.

### 2.3 Loss of endothelial cell identity in the lungs of aged mice following bleomycin challenge

**FIGURE 3:**
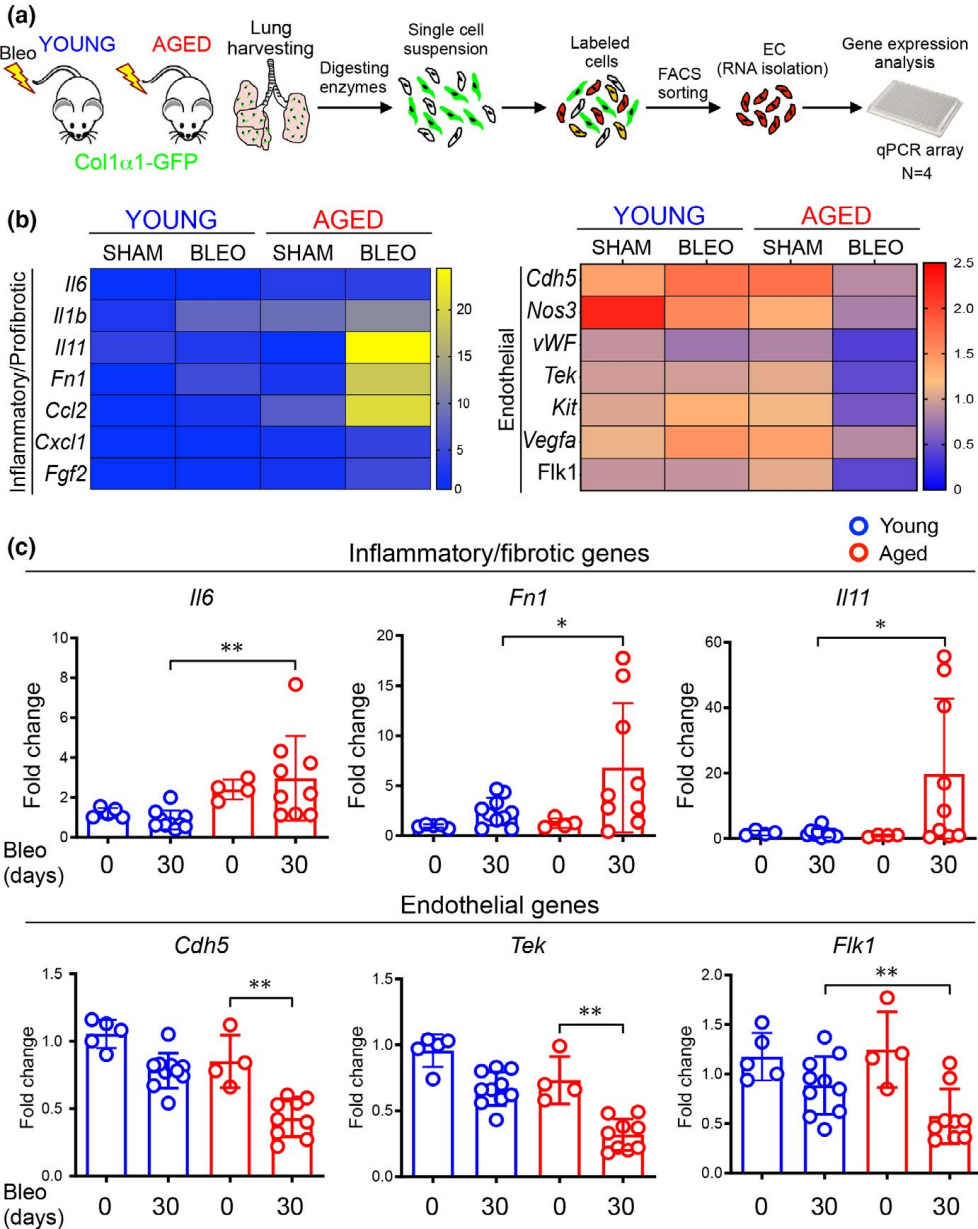
Loss of endothelial cell identity in the lungs of aged mice following bleomycin challenge. (a) Young and aged mice were exposed to bleomycin and sacrificed after 30 and 75 days. Lungs were harvested and prepared for FACS sorting. (b) FACS‐sorted CD31+/GFP−/CD45−/EpCAM− lung ECs from young and aged mice (30 days) were analyzed by using an endothelial cell biology profiler PCR Array (*N* = 4 mice). The heatmap was generated by averaging *n* = 4 mice for each condition. Sham animals were harvested at the same time of bleomycin‐treated animals. The data represent fold changes relative to the young sham and normalized to the housekeeping gene *Actb*. (c) Transcriptional analysis of FACS‐sorted CD31+/GFP−/CD45−/EpCAM− lung ECs isolated from young and aged mice after bleomycin challenge (young sham, *N* = 5, young 30 days, *N* = 10; aged sham, *N* = 4; aged 30 days, *N* = 9). Data passed D’Agostino and Pearson omnibus or Kolmogorov–Smirnov normality test, are expressed as mean ± *SD*, and analyzed using one‐way analysis of variance (followed by Tukey's post hoc test) (*p < 0.05; **p < 0.01; ***p < 0.001).

The limited angiogenic capacity of aged lung and the reduced vascular density observed in the injured lungs of these animals prompted us to further investigate potential mechanisms responsible for this altered vascular behavior. In order to do that, and to capture gene expression signatures that lead to resolution that may be lost at later timepoints, we freshly isolated lung ECs using FACS from both young (*N* = 4) and aged mice (*N* = 4) at 30 days following bleomycin challenge. Next, we measured expression of endothelial genes by using a commercially available qPCR endothelial biology array (Figure 3a,b). We found that lung ECs isolated from aged mice following injury lose their identity, exhibiting a widespread reduction of endothelial cell markers relative to ECs from young mice, including genes known to play role in angiogenesis. In contrast, lung ECs isolated from aged mice following injury exhibited an increased expression of inflammatory and pro‐fibrotic genes, including those encoding for cytokines highly expressed in fibrotic lung, such as IL6 and IL11 (Le et al., 2014; Ng et al., 2019). Gene expression changes of selected genes from the array were further confirmed by qPCR on a larger number of samples as shown in Figure 3c.

All together, these data demonstrated a striking divergence in the endothelial gene expression program that emerges following injury in young and aged mice, with lung ECs from aged mice acquiring an altered transcriptional state reminiscent of that observed during the endothelial/mesenchymal transition (EndMT) (Piera‐Velazquez & Jimenez, 2019). These observations suggest that endothelial injury responses during aging may be a critical driver of progressive lung fibrosis and that reestablishing normal endothelial homeostatic and repair programs may provide a therapeutic strategy to promote fibrosis resolution.

### 2.4 Loss of eNOS leads to sustained lung fibrosis in young animals following bleomycin challenge

**FIGURE 4:**
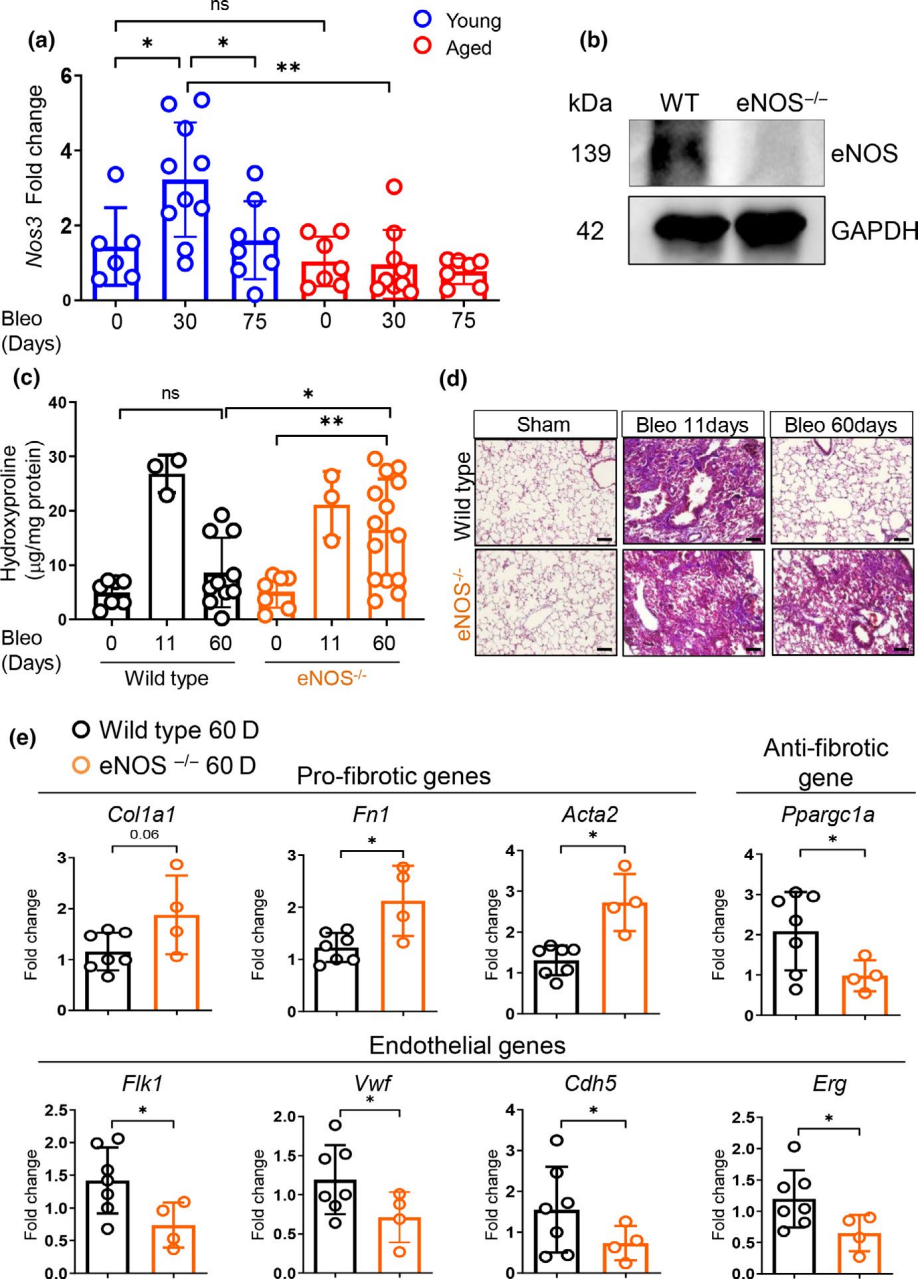
Loss of eNOS leads to sustained lung fibrosis in young animals following bleomycin challenge. (a) *Nos3* transcriptional analysis of FACS‐sorted CD31+/GFP−/CD45−/EpCAM− lung ECs isolated from young and aged mice after bleomycin‐induced lung injury (young sham, *N* = 6; young 30 days, *N* = 10; young 75 days, *N* = 8; aged sham, *N* = 7; aged 30 days, *N* = 9; aged 75 days, *N* = 7). Data passed Kolmogorov–Smirnov normality test, are expressed as mean ± *SD*, and analyzed using one‐way analysis of variance (followed by Tukey's post hoc test). (b) Lung homogenates from WT and eNOS^−/−^ mice were analyzed via Western blot using anti eNOS and anti GAPDH antibodies. (c) Hydroxyproline assay was used to evaluate collagen deposition in the lungs (WT sham, *N* = 7; WT 11 days, *N* = 3; WT 60 days, *N* = 10; eNOS^−/−^ sham, *N* = 7; eNOS^−/−^ 11 days, *N* = 3; eNOS^−/−^ 60 days, *N* = 14). Data passed Shapiro–Wilk normality test, are expressed as mean ± *SD*, and analyzed using one‐way analysis of variance (followed by Tukey's post hoc test). (d) Masson's trichrome assay was used to stain lung tissue. (e) Transcriptional analysis of whole lung homogenates obtained from WT and eNOS^−/−^ mice (WT 60 days, *N* = 7; eNOS^−/−^ 60 days, *N* = 4). The reference group throughout all the genes analyzed in this panel is WT 60 days after bleomycin. Data passed Kolmogorov–Smirnov normality test, are expressed as mean ± *SD*, and analyzed using Student's *t* test (*p < 0.05; **p < 0.01).

Our endothelial gene expression analysis of injured young and aged lungs revealed that several genes involved in vascular remodeling and angiogenesis were significantly reduced in lung ECs from aged mice. Among these was nitric oxide synthase 3 (*Nos3*, encoding endothelial NOS, eNOS), eNOS is predominantly expressed in ECs and belongs to a family of enzymes catalyzing the production of nitric oxide (NO), a gaseous molecule that binds and activates the receptor soluble guanylate cyclase (sGC) in multiple cell types including fibroblasts (Lambers et al., 2019). Previous studies have reported that stimulation of sGC pathway is beneficial in experimental models of organ fibrosis including liver, kidney, heart, and skin (Geschka et al., 2011; Knorr et al., 2008; Stasch, Schlossmann, & Hocher, 2015; Wang et al., 2006). Thus, we hypothesized that absence of enhanced eNOS expression in lung ECs from aged mice may contribute to the sustained fibroblast activation and reduced fibrosis resolution we observed previously (Figure 1). To shed new light on the role of vascular eNOS during lung fibrosis resolution, we first confirmed lower *Nos3* gene expression in freshly isolated lung ECs from a larger cohort of young and aged mice after bleomycin injury. In young mice, we observed that *Nos3* gene expression was significantly elevated at 30 days and returned to baseline at 75 days post‐bleomycin (Figure 4a). In contrast, ECs from aged mice showed no increase in *Nos3* transcript level at the same timepoints. To directly test the role of eNOS during lung fibrosis resolution, we induced lung injury with bleomycin in young eNOS^−/−^ and WT mice and evaluated lung fibrosis by measuring hydroxyproline content and pro‐fibrotic gene expression. Body weight and survival curves showed a modest but significant reduction of weight loss and, although not significant, an increased survival in eNOS^−/−^ relative to WT mice (Figure S1). As shown in Figure 4c, lung hydroxyproline content was comparable in WT and eNOS^−/−^ mice at day 11 post‐bleomycin. However, WT and eNOS^−/−^ mice exhibited divergent resolution responses at later timepoints, with lung hydroxyproline content returning to baseline at day 60 in WT mice but remaining significantly elevated in lungs from eNOS^−/−^ mice at the same timepoint. Lung histological examination confirmed increased collagen in the lungs of bleomycin‐treated eNOS^−/−^ mice at day 60 (Figure 4d).

To evaluate whether the lack of vascular eNOS altered fibrogenic gene expression in the lung following bleomycin challenge, we measured the expression of a panel of fibrotic and anti‐fibrotic genes, including the mitochondrial regulator *Ppargc1a* whose repression is critical during the transition of fibroblasts from a quiescent to an activated state (Caporarello et al., 2019). qPCR analysis of whole lung post‐bleomycin revealed increased pro‐fibrotic gene expression, including *Col1a1*,* Fn1*,* and Acta2*, and a reduction of the anti‐fibrotic gene *Ppargc1a* in eNOS^−/−^ mice compared to WT animals (Figure 4e). Interestingly, injured lungs from eNOS^−/−^ mice also showed reduced expression of the endothelial cell markers *Flk1*,* Vwf*,* Cdh5*,* and Erg* relative to lungs from WT animals (Figure 4e), recapitulating the altered transcriptional responses observed in lung ECs from aged mice post‐bleomycin treatment. All together, these results demonstrate that vascular eNOS plays a critical role during the resolution of bleomycin‐induced lung fibrosis.

### 2.5 eNOS promotes lung fibroblast deactivation through the engagement of the NO/sGC pathway

**FIGURE 5:**
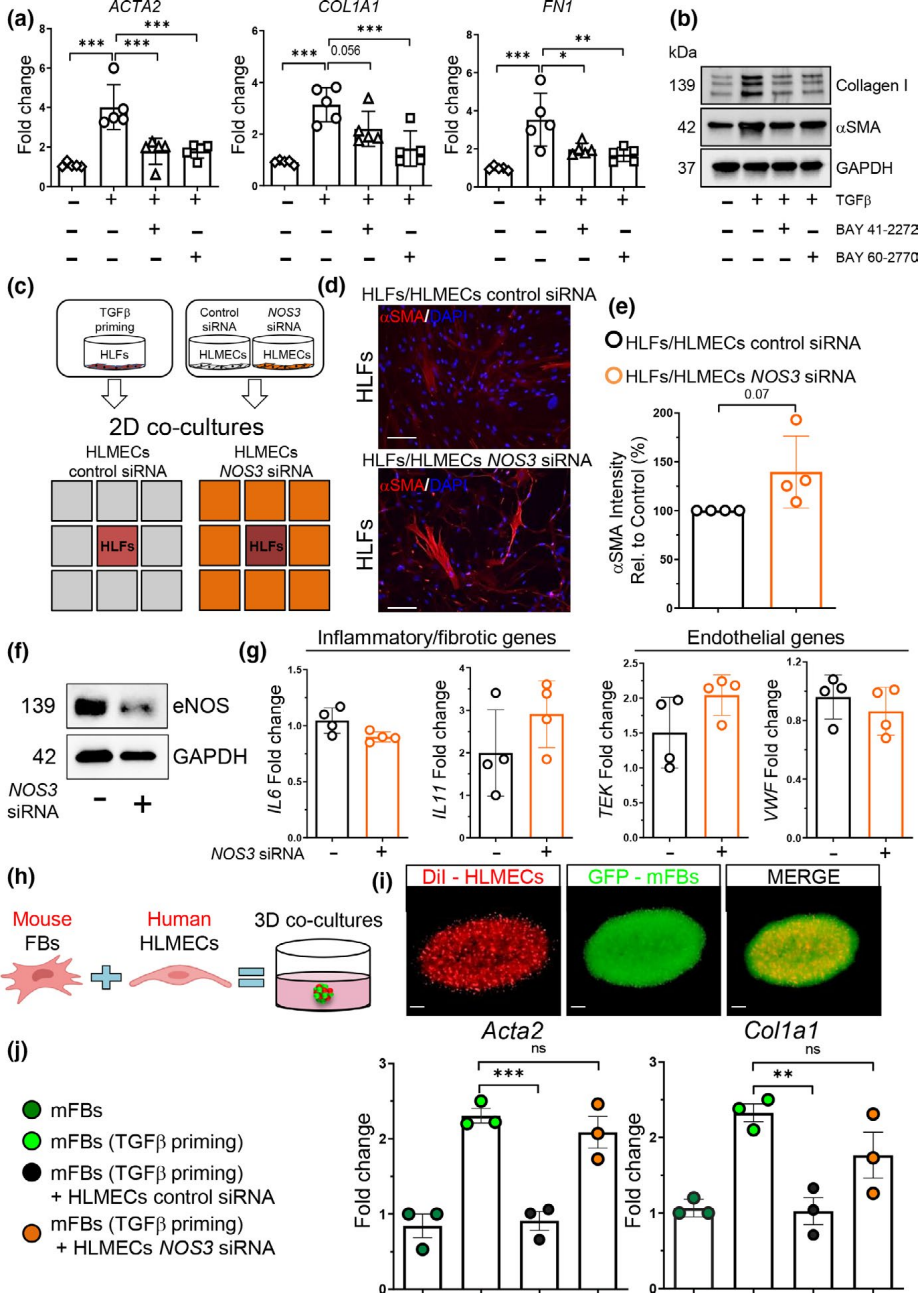
eNOS promotes lung fibroblast deactivation through activation of the NO/sGC pathway. (a, b) Pro‐fibrotic gene and protein analysis of HLFs treated with TGFβ (2 ng/ml) and BAY 41‐2272 (5 μM) or TGFβ (2 ng/ml) and BAY 60‐2770 (1 μM) for 48 hr. *N* = 5 independent experiments. Data passed Kolmogorov–Smirnov normality test, are expressed as mean ± *SD*, and analyzed using one‐way analysis of variance (followed by Tukey's post hoc test). (c) Schematic of 2D co‐culture system. TGFβ‐primed HLFs and control‐ or *NOS3*‐silenced HLMECs were seeded for co‐cultures in μ‐Slide 2 well Co‐culture. (d, e) Immunofluorescence images (10x objective magnification) of HLFs primed 24 hr with 2 ng/ml TGFβ and then co‐cultured with control or *NOS3* siRNA transfected HLMECs (72 hr). Scale bars: 1000 μm. αSMA intensity was determined using automated imaging software. *N* = 4 independent experiments. Data are non‐normally distributed, are expressed as median and IQR, and analyzed using non‐parametric Mann–Whitney test. (f) Control‐ and *NOS3*‐silenced HLMECs (72 hr) were analyzed via Western blot using anti eNOS and anti GAPDH antibodies. (g) Transcriptional analysis of control and *NOS3*‐silenced HLMECs (72 hr). *N* = 4 independent experiments. Data passed Kolmogorov–Smirnov normality test, are expressed as mean ± SD, and analyzed using Student's *t* test. (h, i) Schematic of 3D co‐cultures generation. Visualization of DiI‐labeled HLMECs (red) and Col1α1‐GFP mouse fibroblasts (green) within an endothelial cell fibroblast 3D co‐culture. Scale bar: 500 μm. (j) Gene expression analysis of fibroblasts transcripts from mouse fibroblasts alone versus co‐cultures with control‐ and *NOS3* siRNA transfected HLMECs at day 3. *N* = 3 independent experiments. Data passed Shapiro–Wilk normality test, are expressed as mean ± *SD*, and analyzed using one‐way analysis of variance (followed by Tukey's post hoc test). (**p < 0.01; ***p < 0.001).

NO released from vascular ECs can activate sGC in other cell types, including smooth muscle cells, in a paracrine manner (Kollau et al., 2018). Activation of sGC has been shown to be beneficial in multiple pathological conditions and small molecule activators/stimulators of this pathway are currently being used in the clinic to treat patients with pulmonary hypertension (Ghofrani & Grimminger, 2009). To investigate the contribution of sGC activation in inhibiting pro‐fibrotic gene expression, we treated human lung fibroblasts (HLFs) with TGFβ for 48 hr in the presence or absence of the sGC stimulator BAY 41‐2272 or the sGC activator BAY 60‐2770. As shown in Figure 5a,b, both compounds significantly reduced TGFβ‐induced *ACTA2*, *COL1A1*, and *FN1* expression at RNA and protein levels. Figure 5a,b compares to HLFs treated with TGFβ alone.

To test the ability of ECs to deactivate fibroblasts through the eNOS pathway, we co‐cultured TGFβ‐primed HLFs with human lung microvascular endothelial cells (HLMECs) that have been previously treated with a siRNA targeting eNOS. As shown in Figure 5d,e, TGFβ–primed HLFs co‐cultured for 3 days with eNOS‐silenced HLMECs showed a trend upwards in αSMA intensity compared to HLFs that were co‐cultured with HLMECs transfected with a control siRNA. Analysis of inflammatory/fibrotic (*IL6* and *IL11*) and endothelial (*TEK* and *VWF*) transcripts showed no differences in *NOS3*‐silenced HLMECs compared to control cells (Figure 5g). Because NO disperse rapidly and has a very short half‐life (Thomas, Liu, Kantrow, & Lancaster, 2001), its ability to deactivate fibroblasts may be limited by the distance between cells. To overcome this limitation and to further confirm the capacity of NO to influence pro‐fibrotic gene expression in lung fibroblasts, we adapted a 3D culture system in which cells are mixed within the same matrix (Tan, Choi, Sicard, & Tschumperlin, 2017). FACS‐sorted Col1α1‐GFP mouse lung fibroblasts were primed with TGFβ for 24 hr and subsequently co‐cultured with eNOS‐silenced HLMECs or control silenced cells in a 3D matrigel gel for additional 72 hr. To identify fibroblast‐specific transcriptional changes in our 3D co‐culture system, we designed primers that recognized mouse‐specific sequences in transcripts for analysis by qPCR (Table 1). As shown in Figure 5j, mouse lung fibroblasts cultured with eNOS‐silenced HLMECs showed a significant increase in *Acta2* and *Col1a1* gene expression compared to those cultured with control HLMECs. All together, these findings demonstrated that ECs have the capacity to alter fibroblast activation and promote their deactivation in an eNOS‐dependent manner, suggesting that the absence of induced *NOS3* expression in the lungs of aged mice following injury may directly contribute to the perpetuation of fibroblast activation and fibrosis progression observed in the aging lung.

**Table 1:** Mouse and human primer sequences for qPCR analysis

Primers	Forward	Reverse
*Gapdh*	GTGGAGTCATACTGGAACATGTAG	AATGGTGAAGGTCGGTGTG
*Col1a1*	CCA GCG AAG AAC TCA TAC AGC	GGA CAC CCC TTC TAC GTT GT
*Acta2*	GAGAAGCCCAGCCAGTCG	CTCTTGCTCTGGGCTTCA
*Il6*	TAGTCCTTCCTACCCCAATTTCC	TTGGTCCTTAGCCACTCCTTC
*Fn1*	TGTCAGTCAAAGCAAGCCCG	TTAGGACGCTCATAAGTGTCACCC
*Il11*	AAATTCCCAGCTGACGGAGATCAC	TACATGCCGGAGGTAGGACATCAA
*Erg*	CCGGATACTGTGGGGATGAG	TCTGCGCTCATTTGTGGTCA
*Vwf*	TGTTCATCAAATGGTGGGCAGC	ACAGACGCCATCTCCAGATTCA
*Flk1*	CAAACCTCAATGTGTCTCTTTGC	AGAGTAAAGCCTATCTCGCTGT
*Cdh5*	GTCGATGCTAACACAGGGAATG	AATACCTGGTGCGAAAACACA
*Nos3*	GGCTGGGTTTAGGGCTGTG	CTGAGGGTGTCGTAGGTGATG
*Ppargc1a*	CCCTGCCATTGTTAAGAC	TGCTGCTGTTCCTGTTTT
*GAPDH*	GGAAGGGCTCATGACCACAG	ACA GTC TTC TGG GTG GCA GTG
*ACTA2*	GTGAAGAAGAGGACAGCACTG	CCCATTCCCACCATCACC
*COL1A1*	AAGGGACACAGAGGTTTCAGTGG	CAGCACCAGTAGCACCATCATTTC
*FN1*	TGTCAGTCAAAGCAAGCCCG	TTAGGACGCTCATAAGTGTCACCC

## 3 DISCUSSION

IPF is characterized by the excessive accumulation of extracellular matrix that leads to distortion of lung architecture and loss of organ function. Myofibroblasts are the main source of ECM in IPF lungs and their sustained pathological activation is primarily responsible for the progressive worsening of the disease (Moore & Herzog, 2013). Dysregulated epithelium/mesenchyme cross talk, unresolved inflammation, and limited lung regenerative capacity are among the causes thought to lead to the sustained myofibroblast activation and consequently unresolved fibrosis (Wolters, Collard, & Jones, 2014). In this regard, little is known about the possible link between aberrant fibrosis resolution and vascular remodeling, and elucidation of this interaction is essential to better understand lung repair and regeneration in the context of sustained lung fibrogenesis. Moreover, since aging is associated with endothelial dysfunction (Jane‐Wit & Chun, 2012) and increased risk and severity of fibrotic diseases (Meiners et al., 2015) understanding how vascular alterations during aging contribute to persistent lung fibrosis may lead to novel therapeutic approaches.

In order to identify altered molecular pathways in the pulmonary vasculature that may contribute to sustained fibrosis, we evaluated the responses of lung ECs and fibroblasts to bleomycin‐induced lung injury and fibrosis in young and aged mice. By combining FACS sorting, gene expression, and imaging analysis on injured lungs from both young and aged mice, we demonstrated that lung fibroblast activation in young mice post‐bleomycin is transient and is accompanied by a significant increase in vessel density. On the contrary, fibroblasts isolated from aged lungs post‐bleomycin exhibited a persistent fibrogenic behavior characterized by the sustained elevation of *Col1a1* expression. The lungs of aged mice were characterized by persistently elevated levels of hydroxyproline and significant reductions in lung vascular density. Interestingly, vascular rarefaction has been reported in multiple other mouse models of fibrosis in young mice, including scleroderma and kidney fibrosis (Loganathan et al., 2018; Trojanowska, 2010). Additionally, it has been shown that the loss of capillaries observed in these fibrosis models leads to secondary events including tissue hypoxia and oxidative stress which further exacerbate fibrosis progression by promoting proliferation and activation of tissue resident fibroblasts (Basile et al., 2011; Fleming et al., 2008). Our findings are in part consistent with these previous observations, but only in aged mice. In contrast, bleomycin‐treated lungs from young animals showed a significant increase in vessel density at 30 days, suggesting a potential active role for the lung vascular bed during fibrosis resolution. Intriguingly, the capillary rarefaction we observed in the injured lungs from aged mice is consistent with the non‐resolving nature of this animal model of fibrosis, strongly suggesting that aging may lead to a progressive decline of lung endothelial cell functions thereby influencing fibrosis resolution following lung injury.

Moreover, our ex vivo results from mouse lung explants are consistent with the in vivo observations and demonstrated that while lung explants from young mice responded to angiogenic stimuli and generated numerous vessel sprouts, lung explants isolated from aged mice are refractory to angiogenesis and showed a limited number of microvessels upon angiogenic stimulation. These observations support the notion that lung ECs from aged mice have an impaired angiogenic capacity compared to those from young animals, potentially impacting lung fibrosis resolution following injury. Because fibrotic lungs are characterized by the accumulation of extracellular matrix and by an increased stiffness, a limited angiogenic potential due to an impaired endothelial cell migration through a stiff microenvironment may be responsible for the altered vascular remodeling in injured aged lungs.

In addition, we found that lung ECs isolated from aged lungs after injury have a significant reduction in endothelial markers and an enrichment in inflammatory and fibrotic markers, which is consistent with the transcriptional switch observed during EndMT, a process observed in several chronic conditions including atherosclerosis, pulmonary hypertension, and organ fibrosis (Gong, Lyu, Wang, Hu, & Zhang, 2017; Kitao et al., 2009). During this process, endothelial‐specific markers are lost while mesenchymal and inflammatory markers are acquired, together with alterations in cellular morphology and functions, including loss of the ability of endothelial cells to organize in vessel‐like structures (Piera‐Velazquez & Jimenez, 2019). Moreover, TGFβ, which is highly abundant in fibrotic lungs (Yue, Shan, & Lasky, 2010), plays an important role during EndMT (Piera‐Velazquez & Jimenez, 2019), and *in vitro* evidence indicates that VEGF, whose levels are reduced in IPF patients (Barratt, Flower, Pauling, & Millar, 2018), can block this effect (Yang, Wylie‐Sears, & Bischoff, 2008). Tissue hypoxia is also a critical driver of EndMT (Zhang et al., 2018) and the vascular rarefaction we observed in injured aged lungs could certainly lead to the formation of a hypoxic microenvironment which can influence both endothelial cell fate and consequently fibroblast activation. Interestingly, in line with our results, altered endothelial cell activation and abnormal vascular remodeling also occurs in an experimental model of persistent lung fibrosis which is triggered by repetitive doses of bleomycin (Cao et al., 2016). Hence, our findings suggested that aging leads to the formation of an unfavorable lung microenvironment that promotes vascular rarefaction and endothelial dysfunction thereby influencing disease progression.

Nitric oxide plays an important role in numerous biological processes, including regulation of vascular tone, endothelial cell barrier preservation, endothelial cell survival, and apoptosis (Dimmeler & Zeiher, 1999). Interestingly, it has been reported that endogenous NO plays a protective role in a murine model of experimental lung fibrosis in young mice (Noguchi et al., 2014). In addition, alterations of eNOS pathway have been reported during aging (Cau, Carneiro, & Tostes, 2012). Our transcriptional analysis on freshly isolated lung ECs demonstrated upregulation of Nos3 in young mice at 30 days following bleomycin delivery. In contrast, endothelial cells from aged mice failed to upregulate Nos3 at the same timepoint, strongly suggesting that eNOS may be required for promoting lung fibrosis resolution post‐injury and failure to activate this pathway in the lungs of aged mice contributes to sustained fibrogenesis. Interestingly, in line with our results in the absence of injury, a recent study using single‐cell RNA sequencing reported no differences in lung endothelial *Nos3* expression in aged *vs* young mice (Angelidis et al., 2019). However, single‐cell RNA sequencing data for *NOS3* expression in endothelial cells of IPF versus normal lungs were more variable. In agreement with our results, *NOS3* expression in arterial endothelial cells of IPF lung was reduced; however, an elevation or no differences in other vascular cell types were found (Adams et al., 2019), suggesting that regulation of *NOS3* during disease may occur differently across the endothelial cell types. Together, our observations shed light on the active role of eNOS pathway in promoting lung fibrosis resolution in young mice that is lost with aging. These observations are in line with other studies reporting a protective role for eNOS in experimental models of cardiac and renal fibrosis (Kazakov et al., 2013; Nakayama et al., 2009).

The increased capillary loss we observed in the lungs of aged mice and the lack of transcriptional activation of *Nos3* gene in aged ECs leads us to hypothesize that eNOS/NO pathway may play a critical role in promoting endothelial cell survival following lung injury. Interestingly, previous studies have reported that NO can protect ECs from apoptosis through multiple mechanisms, including blocking caspase activation (Kim, Kwon, Chung, & Kim, 2002) and by promoting survival signaling (Dimmeler & Zeiher, 1999). Our observations together with previous reports suggest that the reduced NO availability in the lungs of aged mice during the resolution phase of bleomycin‐induced lung injury may limit endothelial cell survival thereby contributing to the sustained lung fibrosis in these animals. Another intriguing aspect related to the beneficial effect of NO is its capability to suppress the nucleotide‐binding domain and leucine‐rich repeat containing family, pyrin domain containing 3 (NLRP3) inflammasome in lungs (Mishra et al., 2013). NLRP3 inflammasome activation is implicated in the pathogenesis of IPF (Lasithiotaki et al., 2016) and contributes to the development of experimental lung fibrosis in aged mice, with bleomycin‐treated aged NLRP3^−/−^ mice showing reduced lung fibrosis compared to their WT age‐matched counterparts (Stout‐Delgado et al., 2016). Thus, we speculate that NO‐NLRP3 cross talk may be another anti‐fibrotic mechanism in lung that is lost with aging.

While the role of NO/sGC in regulating vascular tone by promoting smooth muscle cells (SMCs) relaxation has been investigated in great depth (Park et al., 2019; Tsai & Kass, 2009), its contribution to lung fibroblast biology and ECM remodeling has just begun to emerge (Dunkern, Feurstein, Rossi, Sabatini, & Hatzelmann, 2007; Lambers et al., 2019). Our findings demonstrate that in addition to inhibiting αSMA expression, activation of sGC signaling pathways in lung fibroblasts greatly reduces TGFβ responses, including collagen synthesis. Given that ECs can influence the lung microenvironment by producing and releasing angiocrine factors, in this study, we wanted to determine whether lung ECs can directly alter fibroblast activation. Our in vitro results strongly support a functional role for ECs in facilitating the return of activated fibroblasts to a less activated state. While additional work is needed to fully understand endothelial/mesenchymal interactions during lung fibrosis, our work highlights the concept that the lung vascular bed plays an active role during lung fibrosis resolution through the release of NO and the paracrine activation of sGC pathway in neighboring fibroblasts.

A limitation of our study is that we have not measured endogenous NO production or evaluated regulation of eNOS phosphorylation in the lung of young relative to aged mice. Moreover, our results raised several questions not addressed in this study. Investigations aimed at understanding whether restoring *Nos3* expression in aged animals may have beneficial effects in reestablishing endothelial homeostasis and limiting lung fibrosis may be an interesting future subject of study. While our study highlighted the important role of eNOS/NO/sGC during lung fibrosis resolution, a clinical trial conducted in IPF patients showed that sildenafil was not effective for improving pulmonary fibrosis (Kolb et al., 2018). However, sildenafil is a phosphodiesterase‐5 inhibitor that prevents the degradation of cGMP. Importantly, such a drug relies on a sufficient source of endogenous NO to be effective. In contrast, targeting the same pathway to generate cGMP through the use of sGC modulators could be a more suitable therapeutic intervention for treatment of IPF.

Our observations set the stage for future investigations aimed at identifying additional endothelial‐derived signals lost with aging that can influence fibroblast activation as well as the integrity of other neighboring cells, for instance epithelial cells. In fact, recent observations highlighted an important role for ECs in creating a niche for epithelial cells that protect from cell injury and promote repair (Cao et al., 2016; Murray et al., 2017) and aging could contribute to the loss of these pro‐regenerative signals, exacerbating the disease.

In conclusion, our study provides compelling evidence of the direct contribution of the pulmonary vascular bed during lung repair and fibrosis resolution that is lost with aging, suggesting that preserving or rescuing the normal vascular repair and homeostasis responses could represent a new therapeutic strategy to limit lung fibrosis progression.

## 4 EXPERIMENTAL PROCEDURES

Detailed methods are provided in Appendix S1.

### 4.1 Mice

Female and male Col1α1‐GFP transgenic mice (FVB strain) were provided by Dr. Derek Radisky. Female and male wild‐type (C57BL6) and eNOS^−/−^ (Nos3^tm1Unc^/J) mice were provided by Dr. Zvonimir S. Katusic.

### 4.2 Cell culture

Normal primary human lung fibroblasts, HLFs (Walkersville, MD, USA) were used between passages 3 and 7. Normal human lung microvascular endothelial cells, HLMECs (Lonza) were used within passage 4. In experiments involving siRNA, serum was reduced to 0.1%.

### 4.3 Mouse model of bleomycin‐induced lung injury

All animal experiments were carried out conforming to the ARRIVE guidelines. Mice were anesthetized with ketamine/xylazine solution and bleomycin (1 U/kg) or PBS was intratracheally delivered on day 0 as described in Appendix S1.

### 4.4 FACS sorting

Mice were anesthetized with ketamine/xylazine solution and perfused via left ventricle with cold PBS. The lungs were immediately harvested and the single cell suspension was obtained as detailed in Appendix S1. The single cell suspension was incubated with anti‐CD45:PerCp‐Cy5.5 (1:200, Biolegend, Cat# 103132), anti‐CD31:PE (1:200, Biolegend, Cat#102408), anti‐EpCAM:APC (1:200, Biolegend, Cat#118214) antibodies, and DAPI (1:1000, Biolegend, Cat#422801) for 30 min on ice. After incubation, cells were washed with ice‐cold FACS buffer and resuspended in 1 ml of FACS buffer. FACS sorting was conducted using a BD FACS Aria II (BD Biosciences) as described in Appendix S1. Total mRNA was isolated using RNeasy micro kit, followed by Nanodrop concentration and purity analysis. cDNA was synthesized using SuperScript VILO (Thermo Fisher Scientific); RT–PCR was performed using FastStart Essential DNA Green Master (Roche Diagnostics) and analyzed using a LightCycler 96 (Roche Diagnostics).

### 4.5 Fibrosis evaluation

Hydroxyproline content was measured using a hydroxyproline assay kit (Biovision), comparing the samples to a hydroxyproline standard curve as described in Appendix S1.

### 4.6 Immunohistochemistry

Lung tissue from patients with IPF and from non‐fibrotic healthy controls was obtained from Dr. Steven Huang at the University of Michigan. Diagnoses of patients with IPF were established by clinic‐pathologic criteria and confirmed by multidisciplinary consensus conference. All IPF tissues were derived from explanted lungs obtained at the time of transplantation. Normal control lungs were obtained from deceased donors (Gift of Life, Michigan) whose lungs were deemed unsuitable for transplant. All patient samples were obtained with informed consent and were approved by the University of Michigan IRB (IRB #: HUM00105694). Mouse and human lung tissues were stained with Collagen 1α1 (dilution: 1:100; Novus biologicals, Centennial, CO, Cat# NB600‐408) or PECAM‐1 (dilution 1:2000; Abcam, Cat# ab28364) antibodies as described in Appendix S1.

### 4.7 RNA interference

RNA interference was performed with siGENOME Non‐Targeting Control siRNA Pool #1 (D‐001206‐13‐05) or siGENOME Human *NOS3* siRNA (M‐006490‐00‐0005) by using Lipofectamine RNAiMAX reagent (Thermo Fisher Scientific, Cat# 13778075) as described in Appendix S1.

### 4.8 **2D co‐culture and** α**SMA staining**

Co‐cultivation of HLFs and HLMECs and αSMA staining were performed by using μ‐Slide 2 well Co‐culture (ibidi, Lochhamer, Germany). HLFs were primed with TGFβ (2 ng/ml) for 24 hr and then transferred into the inner minor well of the μ‐Slide. HLMECs were transfected with Non‐Targeting or *NOS3* siRNA. Six hours after transfection, the cells were lifted and plated into the outer minor wells of the μ‐Slide. After 72 hr, HLFs were processed and stained as described in Appendix S1.

### 4.9 3D co‐culture generation and analysis

A mixture of FACS‐sorted Col1α1‐GFP mice fibroblasts and HLMECs was plated in a 1:1 solution of Matrigel Matrix (Corning, Cat# 354248) and endothelial cell growth basal medium (Lonza, Cat# 00190860) as detailed in Appendix S1. After 3 days, cells were removed from Matrigel with Corning Cell Recovery Solution (Corning, Cat#35425), and total RNA, cDNA synthesis, and qPCR analysis were performed.

### 4.10 Immunofluorescence staining

Mouse lung tissue sections (7 μm) were permeabilized, blocked, and stained with PECAM‐1 antibody (dilution 1:100; BD Biosciences, Cat# 550274), and nuclei were counterstained with DAPI (dilution: 1:1000; Biolegend, Cat#422801) as described in Appendix S1.

### 4.11 Ex vivo lung tissue culture

Each well of a 48‐well culture plate was coated with 250 μl of rat tail collagen I (2 mg/ml; Thermo Fisher Scientific, Cat# A1048301). Fresh lung explants were embedded into the collagen I layers, cultured for 7 days in presence of VEGFA (20 ng/ml) and analyzed as described in Appendix S1.

### 4.12 Western blotting

Western blotting analysis of whole lung tissue or cell lysates was performed using eNOS (Cell Signaling, Cat#32027) and GAPDH (Cell Signaling, Cat#14C10) antibodies, as described in Appendix S1.

### 4.13 Statistical analysis

Individual data points are shown in all plots and represent data from independent mice or biological replicates from cell culture experiments. Statistical analysis was performed using Student's *t* test, one‐way analysis of variance (followed by Tukey's *post hoc* test), or non‐parametric Mann–Whitney test as detailed in Appendix S1. All analyses and plots were generated using GraphPad Prism 8.0 (La Jolla, CA, USA) with statistical significance defined as *p* < 0.05.

## CONFLICT OF INTEREST

None declared.

## AUTHOR CONTRIBUTIONS

N.C., D.J.T., and G.L. designed the study. N.C., J.A.M., A.A., D.L.J., S.A.A., T. X. P., A.J.H., K.M.C., Q.T., and A.H. performed experiments. N.C. and G.L. analyzed data. The manuscript was drafted by N.C., D.J.T., and G.L. and revised by N.C., S.K.H., Z.S.K., D.J.T., and G.L. All authors participated in manuscript preparation and provided final approval of the submitted work.

## Supporting information

Supplementary Material

## Data Availability

The data that support the findings of this study are available from the corresponding author upon request.
