# Supplementary material for: Vascular dysfunction in aged mice contributes to persistent lung fibrosis
Source: Aging Cell. 2020 Jul 21;19(8):e13196. doi: 10.1111/acel.13196 (PMC7431829; doi:10.1111/acel.13196)
Supplement: Supplementary file 1 — Supplementary Material [file ACEL-19-e13196-s001.docx]

**Vascular Dysfunction in Aged Mice Contributes to Persistent Lung Fibrosis**

Nunzia Caporarello, Jeffrey A Meridew, Aja Aravamudhan, Dakota L Jones, Susan A Austin, Tho X Pham, Andrew J Haak, Kyoung Moo Choi, Qi Tan, Adil Haresi, Steven K Huang, Zvonimir S Katusic, Daniel J Tschumperlin, Giovanni Ligresti

**SUPPLEMENTARY MATERIAL**

**
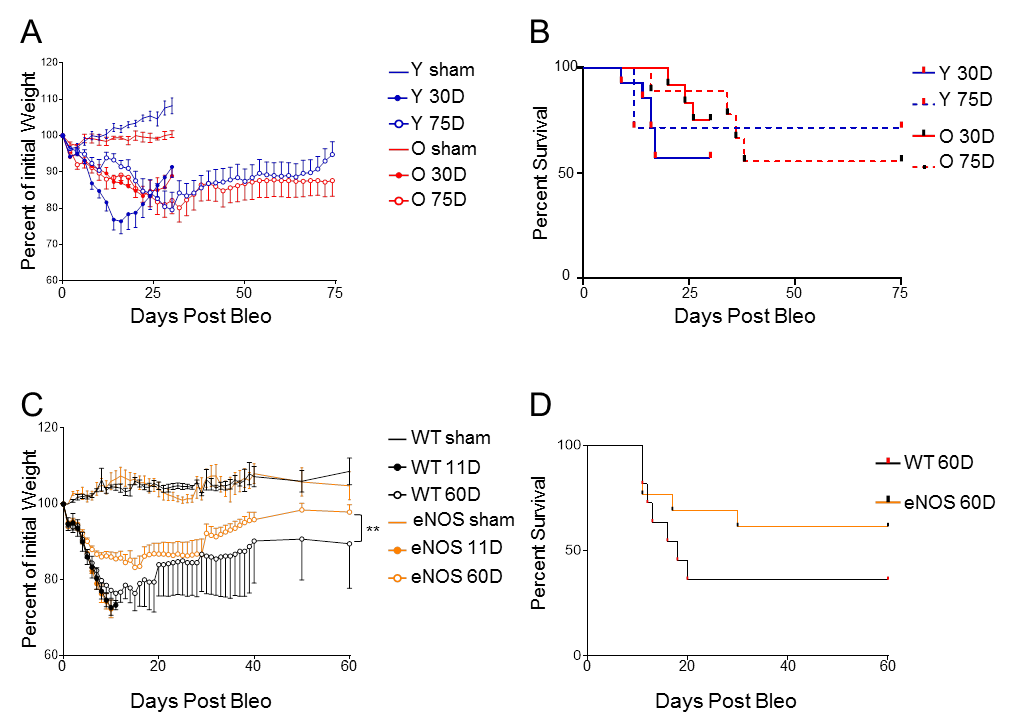
**

**Figure S1. Experimental details for the *in vivo* studies.** **A.** Body weight curves of young and aged animals. Body weights are shown as the percentage of day 0 weight set as 100%. Data are non-normally distributed, are expressed as mean ± SEM and analyzed using non-parametric Welch ANOVA test (young *vs* aged at 30 days, p= 0.8; young *vs* aged at 75 days, p=0.6). **B.** Kaplan-Meier survival curves of young and aged mice. Data are analyzed with Log-rank (Mantel-Cox) test (young *vs* aged at 30 days, p=0.23; young *vs* aged at 75 days, p=0.46). **C.** Body weight curves of WT and eNOS^-/-^ mice. Body weights are shown as the percentage of day 0 weight set as 100%. Data passed D’Agostino & Pearson normality test, are expressed as mean ± SEM and analyzed using ordinary one-way ANOVA test (WT *vs* eNOS^-/-^ at 60 days, p=0.001). **D.** Kaplan-Meier survival curves of WT and eNOS^-/-^. Data are analyzed with Log-rank (Mantel-Cox) test (WT *vs* eNOS^-/-^, p=0.23).

**FULL METHODS**

**Mice**

Female and male Col1α1-GFP transgenic mice (FVB strain) were generated as previously described (UC San Diego, La Jolla, CA) (Yata et al., 2003) and kindly provided by Dr. Derek Radisky. Female and male wild-type (C57BL6) and eNOS ^-/-^ (Nos3^tm1Unc^/J) mice were provided by Dr. Zvonimir S. Katusic. Mice had access to food and water ad libitum and were on a 12h/12h light/dark cycle.

**Cell culture**

Normal primary human lung fibroblasts (HLFs) were purchased from Lonza (Walkersville, MD, USA) and maintained in EMEM (ATCC, Manassas, VA, USA) containing 10% FBS. In experiment involving stimulation of the cells with TGFβ, BAY 41-2272 or BAY 60-2770, serum was reduce to 0.1%. All the experiments were performed with fibroblasts between passages 3 and 7. Normal human lung microvascular endothelial cells (HLMECs) were purchased from Lonza and maintained in endothelial cell growth basal medium (Lonza, Walkersville, MD, USA, Cat # 00190860) supplemented with microvascular endothelial cell growth medium SingleQuots (Lonza, Walkersville, MD, USA, Cat# CC-4147). In experiments involving siRNA, serum was reduced to 0.1 %. All experiments were performed with endothelial cells within fourth passage.

**RNA interference**

Transient RNA interference was performed with siGENOME Non-Targeting Control siRNA Pool #1 (D-001206-13-05) or siGENOME Human *NOS3* siRNA (M-006490-00-0005). Transfection reagent/siRNA complexes were prepared by using Lipofectamine RNAiMAX reagent (Thermo Fisher Scientific, Waltham, MA, USA,) and 10 nmol/L of siRNA in culture medium containing 0.1 % FBS.

**2D co-culture and αSMA staining**

Co-cultivation of HLFs and HLMECs was performed by using μ-Slide 2 well Co-Culture (ibidi, Lochhamer, Germany, Cat# 81806). HLFs were primed with TGFβ (2 ng/ml) for 24 hours, then transferred into the inner minor well of the μ-Slide. HLMECs were transfected with Non-Targeting or *NOS3* siRNA. Six hours after transfection, the cells were lifted and plated into the outer minor wells of the μ-Slide. After cell attachment, the large well of the μ-Slide was filled with EBM2 containing 0.1% FBS to connect the 9 wells and to allow the two cell types to communicate via the supernatant. After 72 hours, HLFs were fixed in 4% formalin for 10 minutes, permeabilized with 0.25% Triton X-100 (Sigma-Aldrich, St Louis, MO, USA), blocked with 1% BSA and then incubated with a Cy3 conjugated αSMA antibody (1:200, Sigma-Aldrich, St Louis, MO, USA, Cat# C6198) for 2 hours at room temperature. Cells were then washed and stained with DAPI (1:1000, Biolegend, San Diego, CA, USA, Cat# 422801). Images were acquired using a Cytation 5 microscope (BioTek, Winooski, VT) with a 10x objective. The cell number for each condition was determined by identifying independent objects on the DAPI channel. αSMA intensity was used to assess the overall protein expression, and was reported relative to cell number, as previously described (Jones et al., 2019). Background was defined as the total intensity observed in an identical well prepared without cells.

**Mouse model of bleomycin-induced lung injury**

All animal experiments were carried out under protocols approved by the Mayo Clinic Institutional Animal Care and Use Committee (IACUC) and conforming to the ARRIVE guidelines. Mouse lung fibrosis was induced with bleomycin (APP Pharmaceutical, LCC Schaumburg, IL, USA, Cat# C103610)as previously described (Haak et al., 2019). Mice were anaesthetized with ketamine/xylazine solution (100 mg/kg, Cat# 0143-9509 and 10 mg/kg, Cat# 59399-110, respectively) and 1 U/Kg bleomycin or PBS was intratracheally delivered using a MicroSprayer (Penn-Century, Philadelphia, PA, USA). Body weight was monitored throughout the study. (*Age study*, young mice. Number at start. Bleo 0, n=5: female; Bleo 30, n=14: female; Bleo 75, n=9: male. Number at end. Bleo 0, n=5: female; Bleo 30, n=8: female; Bleo 75, n=5: male. *Age study,* aged mice. Number at start. Bleo 0, n=7: female; Bleo 30, n=12: female; Bleo 75, n=9: male. Number at end. Bleo 0, n=7: female; Bleo 30, n=9: female; Bleo 75, n=5: male. *eNOS study*, WT mice. Number at start. Bleo 0, n=7: 5 female and 2 male; Bleo 11, n=3: male; Bleo 60, n=24: 9 female and 15 male. Number at end. Bleo 0, n=7: 5 female and 2 male; Bleo 11, n=3: male; Bleo 60, n=10: 6 female and 4 male. *eNOS study*, eNOS^-/-^ mice. Number at start. Bleo 0, n=7: female; Bleo 11, n=3: male; Bleo 60, n=28: 9 female and 19 male. Number at end. Bleo 0, n=7: female; Bleo 11, n=3: male; Bleo 60, n=14: 7 female and 7 male).

**FACS sorting**

Mice were anaesthetized with ketamine/xylazine solution (100 mg/kg, Cat# 0143-9509 and 10 mg/kg, Cat# 59399-110 respectively) injected intraperitoneally and perfused via left ventricle with cold PBS 30 or 75 days after bleomycin or PBS delivery. The lungs were immediately harvested and minced with a razor blade in a 100 mm petri dish in cold DMEM medium containing 0.2 mg/ml Liberase DL and 100 U/ml DNase I (Roche, Indianapolis, IN, USA). The mixture was transferred into 15 ml tubes and incubated at 37 °C for 35 min in a water bath under continuous rotation to allow enzymatic digestion. Digestion was inactivated with DMEM medium containing 10% fetal bovine serum, the cell suspension was passed through a 40 µm cell strainer (Fisher, Waltham, MA, USA) to remove debris. Cells were then centrifuged (1,350 rpm, 10 min, 4 °C), and resuspended in 3 ml red blood cell lysis buffer (Biolegend, San Diego, CA, USA, Cat# 420301) for 90 seconds to remove the remaining red blood cells and diluted in 9mL PBS after incubation. Cells were then centrifuged (1,350 rpm, 10 min, 4 °C) and resuspended in 0.2 ml of FACS buffer (1% BSA, 0.5 mM EDTA pH 7.4 in PBS). The single cell suspension was then incubated with anti-CD45:PerCp-Cy5.5 (1:200, Biolegend, San Diego, CA, USA, Cat# 103132), anti-CD31:PE (1:200, Biolegend, San Diego, CA, USA, Cat#102408), anti-EpCAM:APC (1:200,Biolegend, San Diego, CA, USA, Cat#118214) antibodies and DAPI (1:1000, Biolegend, San Diego, CA, USA, Cat#422801for 30 min on ice. After incubation, cells were washed with ice-cold FACS buffer and resuspended in 1 ml of FACS buffer. FACS sorting was conducted using a BD FACS Aria II (BD Biosciences, San Jose, CA, USA). To isolate CD45-, EpCAM-, CD31-, GFP+ population the following isolation strategy was used: debris exclusion (FSC-A by SSC-A), doublet exclusion (SSC-W by SSC-H and FSC-W by FSC-H), dead cell exclusion (DAPI by PE), CD45 positive cell exclusion (PerCP-Cy5.5 by GFP), EpCAM and CD31 positive cells exclusion (APC by PE), and isolation of GFP positive cells (APC by GFP) as previously described (Ligresti et al., 2019). To isolate CD45-, EpCAM-, GFP-, CD31+ population the following strategy was used: debris exclusion (FSC-A by SSC-A), doublet exclusion (SSC-W by SSC-H and FSC-W by FSC-H), dead cell exclusion (DAPI by PE), CD45 positive cell exclusion (PerCP-Cy5.5 by GFP), EpCAM and GFP positive cells exclusion (APC by GFP), and isolation of CD31 positive cells (APC by CD31). FACS-sorted fibroblasts and ECs were collected in 1.5 ml Eppendorf tubes containing 350 μl of RLT lysis buffer (Qiagen, Valencia, CA, USA) and subjected to mRNA isolation, cDNA synthesis and qPCR analysis.

**Fibrosis evaluation**

Hydroxyproline content was measured using a hydroxyproline assay kit (Biovision, Milpitas, CA, USA, Cat# K555). Briefly, lung samples were transferred into glasses tubes and hydrolyzed with 200 μl 6N HCL at 110°C for 48 hours. The hydrolyzed samples were evaporated to remove excess HCL, reconstituted with 400 μl H_2_O and filtered in 1.5 ml centrifuge tubes equipped with a 0.45 μm semipermeable membrane filter. After samples were added to a 96 well micro-plate, Chloramine T solution was added and the plate was incubated at room temperature for 20 min. 100μl of Erlich’s reagent was added to each well and the plate was incubated at 65°C for 18 min. This method gives an orange red color which is linear up to up to 6 μg of hydroxyproline. OD 550 nm was obtained, and compared to a hydroxyproline standard curve.

**Immunohistochemistry**

Formalin-fixed paraffin embedded (FFPE) lung tissue from patients with IPF and from non-fibrotic healthy controls was obtained from Dr. Steven Huang at the University of Michigan. Diagnoses of patients with IPF were established by clinic-pathologic criteria and confirmed by multidisciplinary consensus conference. All IPF tissues were derived from explanted lungs obtained at the time of transplantation. Normal control lungs were obtained from deceased donors (Gift of Life, Michigan) whose lungs were deemed unsuitable for transplant. All patient samples were obtained with informed consent and were approved by the University of Michigan IRB (IRB #: HUM00105694). Lung tissue from young and aged mice (75 days) were also fixed in formalin and all FFPE blocks were cut in in 7 μm sections and placed on slides. The FFPE sections were deparaffinized using a standard protocol of xylene and alcohol gradients. Sections were then blocked first with BLOXALL endogenous peroxide blocker (Vector Laboratories, Peterborough, UK, Cat# SP-6000) and then with 5% goat serum and 2% BSA (Sigma-Aldrich, St. Louis, MA, USA, Cat# A2153). The staining was performed by using VECTASTAIN Elite ABC HRP kit (Vector Laboratories, Peterborough, UK, Cat# PK-6104), with Collagen 1 alpha 1 primary antibody at 1:100 dilution (Novus biologicals, Centennial, CO, Cat# NB600-408) or PECAM-1 primary antibody at 1:2000 dilution (Abcam, Cambridge, MA, USA, Cat# ab28364). Detection was performed with impact DAB (Vector Laboratories, Peterborough, UK, Cat # SK-4100). Slides were then dehydrated using standard protocol and coverslipped using DPX mountant (Sigma-Aldrich, St. Louis, MA, USA, Cat#06522). Masson’s trichrome staining was performed by using a commercially available stain kit (Abcam, Cambridge, MA, USA, Cat# Ab150686). Quantification of Collagen I in mouse tissue was performed by averaging N=3 non-overlapping fields of view per individual mouse (N=4 mice for each group, young and old 75 days post bleomycin).

**3D co-culture generation and analysis**

Endothelial cell growth basal medium was combined with Matrigel Matrix (Corning, Corning, NY, USA, Cat# 354248) in a 1:1 ratio to produce a 50% Matrigel solution. Each well of a 96 well culture plate was coated with 100 μl of the 50% Matrigel solution. The Matrigel layers were then incubated at 37°C for 45 minutes to allow time for polymerization. FACS sorted Col1α1-GFP mice fibroblasts (GFP+/CD45-/EpCAM-/CD31-) were pretreated with TGFβ (2 ng/ml) for 24 hours in monolayer cultures before to use in 3D co-cultures. Control- or *NOS3* silenced HLMECs were stained with Vybrant DiI Cell-Labeling solution (2 μM, Thermo Fisher Scientific, Waltham, MA, USA, Cat# V22885) for 1 hour before to use in 3D co-cultures. 1x10^5^ mouse fibroblasts and 1x10^5^ HLMECs were suspended in serum free endothelial cell growth basal medium and seeded on the freshly solidified Matrigel layer. For comparisons, 1x10^5^ mouse fibroblasts were suspended alone in the same medium and seeded on the top of the 50% Matrigel layer. After 3 days, cells were removed from Matrigel with Corning Cell Recovery Solution (Corning, Corning, NY, USA, Cat#35425) and total RNA, cDNA synthesis and qPCR analysis were performed as described above.

**Immunofluorescence staining**

Tissue sections (7 μm) from each block were cut in a cryostat at -21 C and mounted onto Vectabond-coated slides (Vector Laboratories, Peterborough, UK). Slides were permeabilized in 0.25 % Triton X-100 (Sigma-Aldrich, St. Louis, MA, USA), blocked with 1% BSA for 1 hour and incubated with an anti PECAM-1 rat primary antibody (BD Biosciences, San Jose, CA, USA, Cat# 550274) diluted 1:100 in PBS with 1% BSA and 5% goat serum, followed by fluorescence-conjugated secondary antibody (Thermo Fisher Scientific, Waltham, MA, USA) and DAPI to counterstain nuclei. Controls were done by omitting primary antibody.

Fluorescence was quantified using a binary histogram threshold to identify fluorescent area and then to calculate relative PECAM-1 density. Measurements of 5 to 11 fields-of-view were taken from each mouse and their average and standard deviation are reported.

The optimal threshold level was determined using in-house code to select the threshold level based on the number of standard deviations above the image mean. The threshold used for this study was 6 standard deviations above the mean and was applied uniformly across all images.

***Ex vivo* lung tissue culture**

Each well of a 48 well culture plate was coated with 250 μl of rat tail collagen I (2 mg/ml, Thermo Fisher Scientific, Waltham, MA, USA, Cat# A1048301) and left on ice to avoid polymerization. Mice were anaesthetized with ketamine/xylazine solution (100 mg/kg, Cat# 0143-9509 and 10 mg/kg, Cat# 59399-110 respectively) injected intraperitoneally and then perfused via left ventricle with cold PBS. Fresh lung explants were cut in small pieces (1-2 mm^2^) and embedded into the collagen I layers, which were then incubatedat 37°C for 30 minutes to allow time for polymerization.. Once embedded, the explants were fed with serum-free EBM supplemented with VEGFA 20 ng/ml. Medium was replaced every 2-3 days. After 7 days, the angiogenic response of the lung explants was measured by manually counting the number of neovessels as previously described (Ligresti, Aplin, Zorzi, Morishita, & Nicosia, 2011).

**Real-time PCR**

Total mRNA was isolated using RNeasy micro kit or minikit (Qiagen, Valencia, CA, USA) followed by Nanodrop concentration and purity analysis. cDNA was synthesized using SuperScript VILO (Thermo Fisher Scientific, Waltham, MA, USA); RT–PCR was performed using FastStart Essential DNA Green Master (Roche Diagnostics, Mannheim, Germany) and analyzed using a LightCycler 96 (Roche Diagnostics, Mannheim, Germany). RT-PCR primers used in this study (Integrated DNA Technologies, Coralville, IA, USA) are listed in Table 1. qPCR Array was performed by using RT^2^ Profiler^TM^ PCR Array Mouse Endothelial Cell Biology (Qiagen, Valencia, CA, USA). For qPCR Array, data represent fold changes relative to the young sham and normalized to the housekeeping gene *Actb*.

**Protein extraction and Western blot analysis**

Cell proteins were extracted using RIPA lysis buffer (ThermoFisher Scientific, Waltham, MA, USA). Protein concentration was determined using Pierce BCA Protein Assay Kit (ThermoFisher Scientific, Waltham, MA, USA). Whole lung tissue or cell lysates were separated by electrophoresis, transferred onto PVDF membranes and incubated overnight at 4 degrees with primary antibodies: eNOS (Cell Signaling, Danvers, MA, USA, Cat#32027) and GAPDH (Cell Signaling, Danvers, MA, USA, Cat#14C10). Blots were then washed and incubated with appropriate secondary antibodies for 1 hour at room temperature. Bands were visualized by using ChemiDoc Imaging System (Bio-Rad, Hercules, CA, USA), according to the manufacturer’s protocol.

**Statistical Analysis**

Individual data points are shown in all plots and represent data from independent mice, cells, or biological replicates from cell culture experiments. Depending on the group size, normality distribution was assessed with D’Agostino-Pearson omnibus, Shapiro-Wilk or Kolmogorov-Smirnov normality tests. Variables with normal distribution are summarized as mean and SD, with statistical comparison between two groups performed using Student’s t-test and comparison of more than two groups performed using one-way analysis of variance (followed by Tukey’s *post hoc* test). Variables with non-normal distribution are summarized as median and IQR, with statistical comparison between two groups performed using non-parametric Mann Whitney test and comparison of more than two groups performed with non-parametric Kruskal-Wallis test (followed by Dunn’s post-test).Weight loss data are presented as mean ±SEM, with statistical comparison between groups performed using non-parametric Welch ANOVA test or ordinary one-way ANOVA test. Survival data are presented by the Kaplan-Meier method, with statistical comparison between groups performed using Log-rank (Mantel-Cox) test. All analyses and plots were generated using GraphPad Prism 8.0 (La Jolla, CA, USA) with statistical significance defined as p < 0.05.

References

Haak, A. J., Kostallari, E., Sicard, D., Ligresti, G., Choi, K. M., Caporarello, N., . . . Tschumperlin, D. J. (2019). Selective YAP/TAZ inhibition in fibroblasts via dopamine receptor D1 agonism reverses fibrosis. *Sci Transl Med, 11*(516). Retrieved from <https://www.ncbi.nlm.nih.gov/pubmed/31666402>. doi:10.1126/scitranslmed.aau6296

Jones, D. L., Haak, A. J., Caporarello, N., Choi, K. M., Ye, Z., Yan, H., . . . Tschumperlin, D. J. (2019). TGFbeta-induced fibroblast activation requires persistent and targeted HDAC-mediated gene repression. *J Cell Sci, 132*(20). Retrieved from <https://www.ncbi.nlm.nih.gov/pubmed/31527052>. doi:10.1242/jcs.233486

Ligresti, G., Aplin, A. C., Zorzi, P., Morishita, A., & Nicosia, R. F. (2011). Macrophage-derived tumor necrosis factor-alpha is an early component of the molecular cascade leading to angiogenesis in response to aortic injury. *Arterioscler Thromb Vasc Biol, 31*(5), 1151-1159. Retrieved from <https://www.ncbi.nlm.nih.gov/pubmed/21372301>. doi:10.1161/ATVBAHA.111.223917

Ligresti, G., Caporarello, N., Meridew, J. A., Jones, D. L., Tan, Q., Choi, K. M., . . . Tschumperlin, D. J. (2019). CBX5/G9a/H3K9me-mediated gene repression is essential to fibroblast activation during lung fibrosis. *JCI Insight, 5*. Retrieved from <https://www.ncbi.nlm.nih.gov/pubmed/31095524>. doi:10.1172/jci.insight.127111

Yata, Y., Scanga, A., Gillan, A., Yang, L., Reif, S., Breindl, M., . . . Rippe, R. A. (2003). DNase I-hypersensitive sites enhance alpha1(I) collagen gene expression in hepatic stellate cells. *Hepatology, 37*(2), 267-276. Retrieved from <https://www.ncbi.nlm.nih.gov/pubmed/12540776>. doi:10.1053/jhep.2003.50067
